# A generative spiking neural-network model of goal-directed behaviour and one-step planning

**DOI:** 10.1371/journal.pcbi.1007579

**Published:** 2020-12-08

**Authors:** Ruggero Basanisi, Andrea Brovelli, Emilio Cartoni, Gianluca Baldassarre

**Affiliations:** 1 Institut de Neurosciences de la Timone UMR 7289, Aix Marseille Université, CNRS, Marseille, France; 2 Institute of Cognitive Sciences and Technologies, National Research Council, Rome, Italy; École Normale Supérieure, College de France, CNRS, FRANCE

## Abstract

In mammals, goal-directed and planning processes support flexible behaviour used to face new situations that cannot be tackled through more efficient but rigid habitual behaviours. Within the Bayesian modelling approach of brain and behaviour, models have been proposed to perform planning as probabilistic inference but this approach encounters a crucial problem: explaining how such inference might be implemented in brain spiking networks. Recently, the literature has proposed some models that face this problem through recurrent spiking neural networks able to internally simulate state trajectories, the core function at the basis of planning. However, the proposed models have relevant limitations that make them biologically implausible, namely their world model is trained ‘off-line’ before solving the target tasks, and they are trained with supervised learning procedures that are biologically and ecologically not plausible. Here we propose two novel hypotheses on how brain might overcome these problems, and operationalise them in a novel architecture pivoting on a spiking recurrent neural network. The first hypothesis allows the architecture to learn the world model in parallel with its use for planning: to this purpose, a new arbitration mechanism decides when to explore, for learning the world model, or when to exploit it, for planning, based on the entropy of the world model itself. The second hypothesis allows the architecture to use an unsupervised learning process to learn the world model by observing the effects of actions. The architecture is validated by reproducing and accounting for the learning profiles and reaction times of human participants learning to solve a visuomotor learning task that is new for them. Overall, the architecture represents the first instance of a model bridging probabilistic planning and spiking-processes that has a degree of autonomy analogous to the one of real organisms.

## Author summary

Goal-directed behaviour relies on brain processes supporting planning of actions based on their expected consequences before performing them in the environment. An important computational modelling approach proposes that the brain performs goal-directed processes on the basis of probability distributions and computations on them. A key challenge of this approach is to explain how these probabilistic processes can rely on the spiking processes of the brain. The literature has recently proposed some models that do so by ‘thinking ahead’ alternative possible action-outcomes based on low-level neuronal stochastic events. However, these models have a limited autonomy as they require to learn how the environment works (‘world model’) before solving the tasks, and use a biologically implausible learning process requiring an ‘external teacher’ to tell how their internal units should respond. Here we present a novel architecture proposing how organisms might overcome these challenging problems. First, the architecture can decide if exploring, to learn the world model, or planning, using such model, by evaluating how confident it is on the model knowledge. Second, the architecture can autonomously learn the world model based on experience. The architecture represents a first fully autonomous planning model relying on a spiking neural network.

## Introduction

In mammals, the acquisition and consolidation of instrumental behaviour involves two sets of processes, one underlying flexible *goal-directed behaviour*, used in particular to find solutions to new problems or to face changing conditions, and the other one related to *habits*, forming stimulus-response behaviour used to efficiently, but inflexibly, face familiar conditions [1–3]. As also highlighted in the computational literature [4], goal-directed behaviour is *model based*; that is, it relies on an internal representation of the external world (in particular of the transition probabilities between its states; the so called *world model*) to internally simulate the consequences of actions, or action sequences, usable to achieve desired world states (*goals*) before executing them in the environment (*planning*) [5–10] (note that here goals are intended as internal representations of desired world states [11], rather than in the broader meaning of world/body states to which the organism homeostatically converges [12]). When the agent pursues a new goal and has a world model to do so, goal-directed processes allow the solution of the task on the basis of planning. Indeed, the world model represents the general goal-independent dynamics of the world, in particular how it responds to the agent’s actions. The simulated achievement of the new goal based on the world model might be possibly marked by an internal reward [13] and to an external observer the agent appears to solve the new task ‘on the fly’ or ‘by insight’. Instead, habitual behaviour is *model free*, in the sense that it relies on *actions directly triggered by stimuli* (*habits*) and does not require a world model anticipating their outcomes [4, 9, 14]. Habits are task dependent as they can only lead to specific desirable world states. Thus, given a new desired state, repeated experience is needed to discover and learn by trial-and-error the new stimulus-response associations leading to it.

In the brain, goal-directed behaviour relies on ventral/associative basal ganglia and pre-frontal cortex areas supporting the representation of goals and the world dynamics; instead, habitual behaviour relies on motor basal ganglia and sensorimotor/premotor cortices able to acquire stimulus-response associations by reinforcement learning [14–18]. The brain processes underlying goal-directed behaviour have been interpreted within different computational frameworks. A current influential view of the brain, rooted in Helmholtz’ pioneering contributions on perception [19], considers it a *probabilistic* or *Bayesian machine* that copes with the uncertainties of the world by representing it in terms of probability distributions and probability inferences on them pivoting on the Bayes rule [20, 21]. This view of the brain has been progressively extended to cover all aspects of cognition, from perception to action and decision making (e.g., [22, 23]). In line with this view, it has been proposed that the brain also implements goal-directed behaviour and planning through probabilistic representations and inferences, and this has been shown through specific models developed within an approach called *planning as inference* (e.g., [24–26]). This approach uses world representations expressed as probability distributions and performs action selection based on a probability inference maximising the expectation of the desired world state (more details in Sec 1).

The models of planning as inference commonly use probability distributions that directly involve high-level aspects of cognition and behaviour, for example observations, world states, and actions; moreover, the inferences on these distributions are based on sophisticated mathematical manipulations of the parameters of the distributions, for example those based on *Hidden Markov Models* (HMMs), or on numerical approximations of them. This gives rise to a fundamental challenge for these models [21, 27–29]: *how can the probability distributions and inference processes supporting goal-directed processes be grounded on the low-level spiking events of neurons in the brain?*

An important possibility is that the needed probability distributions rely on the probability distributions of neuron spikes, sampled by the actual spikes; and that the connections between neural populations, undergoing experience-dependent plasticity, support the conditional probabilities underlying probabilistic inferences [25, 30–34]. In this respect, spikes can be seen as sampling probability distributions analogously to what happens in *particle filters* [35–37]. These are algorithms that use a set of values (‘particles’) to represent the distributions of stochastic processes such as HMMs (particle filters draw a set of random values –the ‘particles’– for each probability distribution to represent, consider the dependencies between different distributions by ‘propagating’ the particles between them, and use value weights and re-sampling mechanisms to approximate complex distributions and take observations into account; [37]). In this respect, the model presented here relies on a general principle, also shared with previous models [38–40], termed here *emergent generativity*. We refer emergent generativity to the process for which the stochastic events of spiking neurons, happening at the micro/low level, are amplified by neural mechanisms to generate alternative cognitive contents, at the macro/high level, that support adaptive behaviour (e.g., alternative possible imagined percepts, believes, and courses of action). This concept is further discussed in Sec 3.2.

Although planning as inference was previously modeled with a firing-rate neural network [41], only recently recurrent spiking neural network models have been used to implement planning as inference [38, 39, 42]. These models, which are the state-of-the-art in the field, use recurrent neural networks to represent the world model. Here different groups of neurons represent different world states, for example different places in a navigation maze, and their lateral connections encode the possible transitions between states that the agent might cause with action. The spikes of the world model sample the prior probability of the state sequences followed by the agent if it explores the environment randomly, and of the rewards associated to the sequence (e.g., a reward of 1 when a target state is achieved). A second neural layer of spiking neurons that encodes the ‘context’, intended as the current and target states, sends all-to-all connections to the world model and can condition the probability distribution it expresses. The neural solution to the inference problem relies on the update of the connections linking the context to the world model so that the distance (Kullback-Leibler divergence) between the prior probability distribution of the state sequences converges to the desired posterior probability distribution maximising the reward. The actions needed to follow the state sequences sampled from the posterior distribution are inferred by inverse kinematics, either offline [38] or using a dedicated neural layer [39]. Another related model [40] reproduces goal-directed behaviour with an analogous recurrent spiking neural network. Here the actions that correspond to a decision-state are reciprocally linked by inhibitory connections to implement decision making. For a given task, reward units ‘inject’ activation into terminal actions, and this activation diffuses backward towards the upstream actions to represent the anticipated value attributed to them. This value is then used for action selection.

These models represent an important first step in modelling how the brain might implement planning as inference, but much remains to be understood since planning in animals involves several interdependent complex processes such as the formation of goals, their motivational activation, the acquisition of world models, the formulation of plans at multiple levels of abstraction, the performance of actions, and the coordination of these different processes [43].

In this work we contribute to face these issues by tackling two important problems not solved by the state-of-the-art models considered above. The first problem is: *how can the brain acquire the world model while at the same time using it for planning?* The model-free literature on reinforcement learning [4] studies the important problem of the exploration-exploitation trade-off where an agent must decide whether to take random actions to explore the environment and learn the policies that lead to rewards, or to exploit those policies to maximize rewards. A problem less studied involves a situation where model-based/goal-directed agents have to face an analogous but different trade-off [44–46]. In particular, when these agents solve new tasks they have to decide if exploring to refine the world model, or if exploiting such model to plan and act. Here we consider the early phases of the solution of new tasks, involving either a new environment or a new goal, and hence focus on the latter type of exploration-exploitation trade-off. This problem has been recently faced in a principled way [46] within the probabilistic framework of active inference [22]. However, the proposed solution is applicable only to very simple scenarios where hidden-states are few and are given to the agent, rather than being autonomously acquired; moreover, and importantly for our objective, the solution has not been grounded on brain-like mechanisms. On the other side, current state-of-the-art models implementing planning as inference based on spiking networks either learn the world model before solving the target task [38, 39] or use a hardwired world model [40], and so they do not face the problem altogether. How the brain manages to learn and use the world model at the same time is hence a fully open problem.

The second problem we face here, not solved by the current planning-as-inference spiking models, is: *how could the brain learn the world model in an unsupervised fashion?* Currently there are no biologically acceptable solutions to this problem as the current state-of-the-art models either learn the world model off-line through supervised learning techniques [38, 39] or are given a hardwired model [40].

Here we propose a model architecture facing both problems limiting the current planning-as-inference spiking-network models. The architecture tackles the first problem by proposing a novel *arbitration mechanism* measuring the uncertainty of the world model on the basis of the entropy of the posterior probability distributions expressed by the neurons forming it (cf. [47, 48]). When this uncertainty is low, planning continues, otherwise exploration actions are performed. Recently, it has been shown that the contextual learning and use-for-planning of the world model encounter a difficult problem for which the world model can prematurely converge to sub-optimal solutions (‘bad-bootstrapping’ problem, [46]).

A second novelty of the architecture is the solution of the second problem. The solution is in turn based on three innovations. First, the integration of the unsupervised STDP learning rule proposed in [49] into the recurrent spiking neural-network world model. This allows the world model to learn at the same time the hidden causes of observations and the probabilistic time dependencies between them. This is a notable advancement in terms of biological plausibility with respect to current models using supervised learning mechanisms that directly activate the internal units to encode hidden causes [38–40]. This also represents a computational advancement as the only recently proposed (non-spiking) probabilistic model tackling the model-based exploration/exploitation problem [46] assumes to know the hidden causes of observations. The second mechanism relies on the idea that the world model is a HMM that ‘observes’, learns, and predicts sequences of items formed not only by percepts but also actions. Actions are in particular ‘observed’ by the world model after being selected by planning or exploration processes. This idea was suggested by evidence indicating that various brain areas receive (‘observe’) both sensory and motor information, for example the parietal cortex [50, 51], the prefrontal cortex [43], and the hippocampus [52]. This, integrated with the third mechanism introduced below, allows the world model to autonomously select actions without the need of an auxiliary component selecting actions on the basis of state sequences (e.g., as in [38]). The third mechanism is based on the conditioning of the posterior probabilities of the world model on the pursued goal. This implies that with no goal conditioning the world model represents the prior probabilities of arbitrary state-action sequences, while when a goal is selected (‘clumped’) the model represents the posterior probabilities directly producing action-sequences leading to the goal.

The model was validated by testing it against the results reported in [15, 16] where human participants learn to solve a visuomotor learning task. In particular validation checked if the learning processes of the world model led to match human performance, and if the planning time spent by the arbitration mechanism reproduced the reaction times exhibited by human participants. The target experiment was also investigated with a model in [48]; however, this model did not aim to bridge planning as inference to spiking network mechanisms. To our knowledge, our model is the first of this type to be validated with specific detailed behavioural data.

The rest of the paper is organised as follows. Section 1 describes the model architecture and functioning and the visuomotor learning task used to validate it. Section 2 presents the results of the model tests, in particular by comparing the model performance and reaction times with those of human participants of the target visuomotor task, and by showing the mechanisms that might underlie such performance. Section 3 discusses such results in the light of the literature. Finally, Section 4 draws the conclusions. Particular attention has been paid to make the paper accessible to a wide interdisciplinary audience, as requested by the journal; this was also facilitated by leveraging the heterogeneous background of the authors.

## 1 Methods

This section first illustrates the task used to test the model [15, 16] and gives an overview of its architecture and functioning. Then it explains the HMMs relevant for this work, the spiking neural network equivalent to a HMM used to implement the world model of the architecture, the arbitration and exploration components of the architecture, and the procedure used to search its meta-parameters. The initial draft of this paper was published in [53].

### 1.1 Target experiment

**Fig 1:**
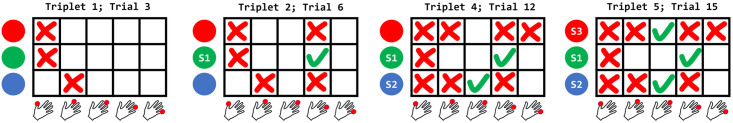
The visuomotor learning task used to validate the model. Three colour stimuli are presented to the participants in a pseudo-random order, in particular in triplets each containing each colour exactly once. The action consists in pressing one out of five possible buttons with the right hand. The figure refers to an ideal participant who never repeats an error for the same colour and always repeats the correct action after discovering it. The four pictures refer to respectively the actions after one, two, four, and five triplets: a red cross and a green tick-mark refer to incorrect and correct colour-action sequences respectively. The colour receiving the first action in the second triplet is marked as the ‘first stimulus’ (S1), and such action is considered the as correct one for it. The colour different from S1 receiving the first action in the fourth triplet is marked as the ‘second stimulus’ (S2), and such action is considered as the correct one for it. The colour different from S1 and S2 receiving the first action in the fifth triplet is marked as the ‘third stimulus’ (S3), and such action is considered the correct one for it.

In the task used to test the model [15, 16], human participants are supposed to discover the correct associations between three different stimuli and three possible motor responses chosen from five possible ones (Fig 1). During the experiment, three different colours are projected on a screen in a pseudo-randomised order, in particular through twenty triplets each involving the three colours (each triplet is thus formed by three ‘trials’). After each colour perception, the participants have to respond by pressing one of five buttons of a keyboard with their right hand. Once this action is performed, a feedback on the screen informs the participants if the association between the colour and the performed action was correct or wrong. The goal of the participants is to obtain a ‘correct’ feedback for each colour by selecting the corresponding ‘correct action’. Unbeknown to the participants, however, the correct action for each colour is not set a-priori but is established dynamically during the experiment to obtain a fixed number of exploration actions for the three colours among the different participants. In particular, for each colour stimulus ‘S’ a fixed number of ‘incorrect feedback’ outcomes are given to the participants before considering the performed action as correct: thus, for S1 a ‘correct’ feedback is given at the second action (hence after one error), for S2 at the fourth action (after three errors), and for S3 at the fifth action (after four errors). The colour stimulus considered as S1, S2, and S3 is itself established dynamically as the first colour, not yet associated to a correct action, presented within respectively the second, fourth, and fifth triplet. Notice that with this procedure the participants are not supposed to explore all the possible colour-action associations but rather to only discover, and then exploit, the colour-action association needed to accomplish the ‘correct feedback’ goal. The task has been designed to differentiate between two phases of the participants’ behaviour: an initial exploration phase where they are expected to search the correct colour-action associations, and a second exploitation phase where they are supposed to repeat the found correct associations until the end of the task.

### 1.2 Goal-directed behaviour model: Overview of the architecture and functioning

#### 1.2.1 Architecture

**Fig 2:**
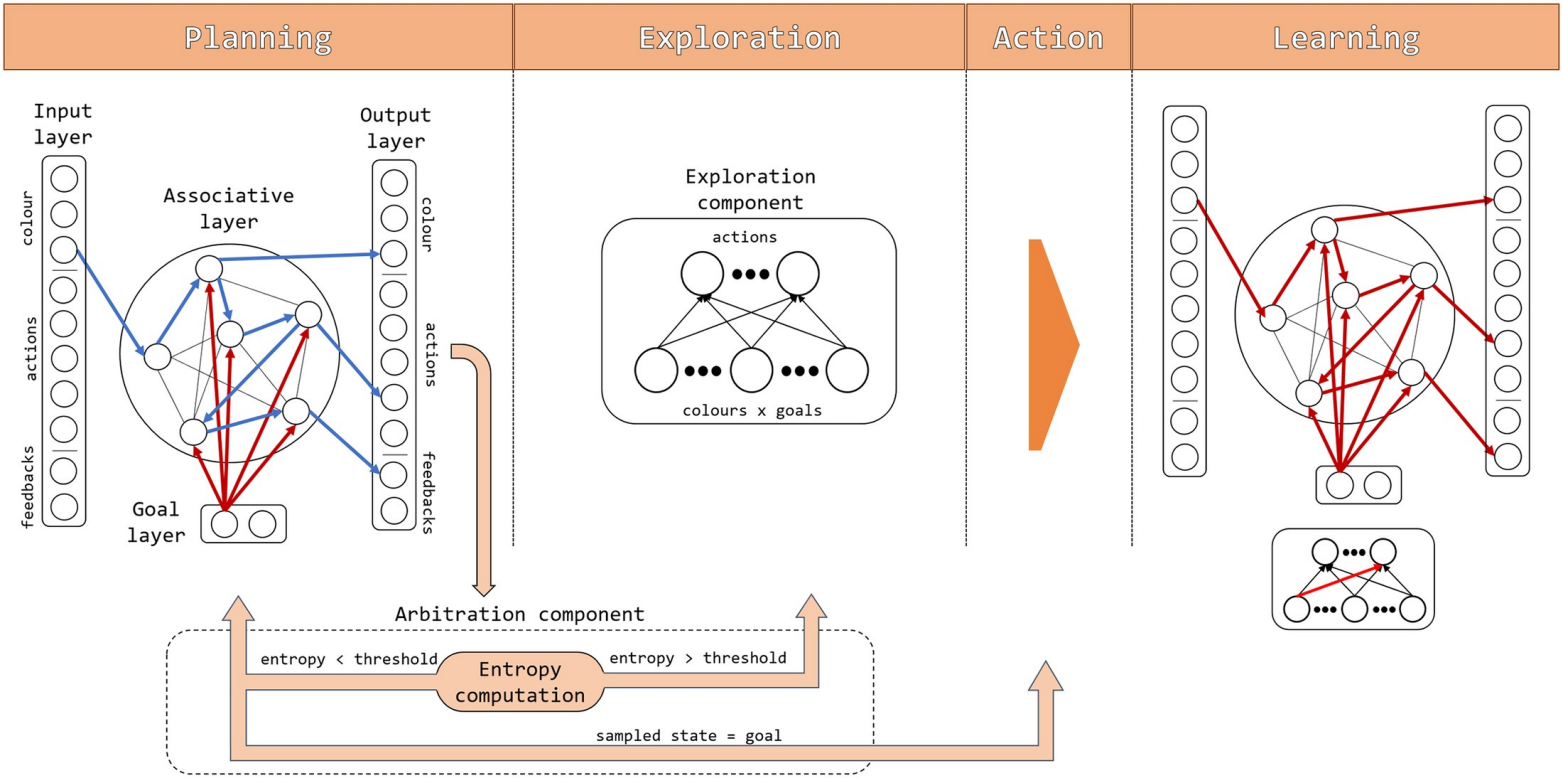
Architecture and functioning of the model: Components and information flow. The architecture is formed by a planning component (representing input patterns, hidden causes of input patterns within an associative layer, expected events including actions, and goals), an exploration component selecting actions when planning is uncertain, and an arbitration component deciding when to plan, explore, or act. The figure also shows the timing of the processes taking place during a trial, with the first two left graphs showing the Planning (exploitation) and (possibly) Exploration phases and the right two graphs showing the Action execution and Learning phases. Blue arrows represent an example of information flow travelling stable connections during the Planning phase and red arrows represent information flows travelling connections that are updated during the Learning phase.

Fig 2 gives an overview of the architecture and functioning of the model. The architecture of the model is composed of a spiking neural network for planning formed by four different layers, a spiking neural network for exploration formed by two neural layers, and a non-neural arbitration component. The four layers forming the core neural network, which supports planning by instantiating a HMM, are now considered more in detail.

*Input layer*. The input layer contains ten neurons, three encoding the stimuli (colours), five encoding the actions (different possible finger presses), and two encoding the outcome (correct or incorrect feedback). The input layer sends all-to-all afferent connections to the neurons of the associative layer.

*Goal layer*. The goal layer is composed of neurons encoding the goals to achieve, here two neurons encoding the two goals of the visuomotor task: ‘obtain a correct feedback’ and ‘obtain an incorrect feedback’ (the use of the latter is explained later). To commit to achieving a certain goal the agent activates the corresponding neuron on the basis of internal mechanisms not simulated here. Goal neurons send all-to-all efferent projections to the associative neurons.

*Associative layer*. The associative layer, forming the core of the model, is composed of 400 neurons, all connected to each other but without self-connections. The associative layer receives the mentioned afferent connections from the input and goal layers, and sends all-to-all efferent connections to the neurons of the output layer.

*Output layer*. As the input layer, the output layer is composed of ten neurons each one representing one of the stimuli, actions, and outcomes of the task. The output layer receives the mentioned afferent connections from the associative layer.

Together the four layers instantiate a neural HMM implementing the world model used for planning. In particular, the input and output layers together form the observation part of the HMM, and have an identical structure. Given that the connections of real neurons are unidirectional, we used the two layers to implement separately the two functions played by the observation part of the HMM, namely the input from the external environment and the possible generative reconstruction of such input based on internal causes. The associative layer encodes the probability distribution over the hidden causes of the observations and the probabilistic temporal dependencies between them. The goal layer can condition the latter distributions to possibly increase, with learning, the probability of sampling simulated colour-action-feedback sequences that lead to the desired goal. An important feature of the HMM implemented by the model is that, as in [49], each of the three events of each trial (colour, action, feedback) is represented by a sequence of active HMM nodes that encode not only one of the events but also the time step when it is present. For example, after learning a certain group of neurons encodes an action and the neurons of the group fire in sequence for a certain number of time steps corresponding to the action duration.

Alongside the planning components, the architecture is formed by the following additional components used for exploration and arbitration.

*Exploration component*. This component is formed by two layers of spiking neurons, namely (a) an input layer encoding the combinations of colours and goals (3 × 2 neurons corresponding to 3 colours and 2 goals), and (b) an output layer encoding the five possible finger-press actions (five neurons).

*Arbitration component*. This component, currently not implemented with neural mechanisms, decides when to plan, explore, or act in the world. The decision is made on the basis of the level of knowledge of the world model, measured as the average entropy of its probability distribution during the last ‘planning cycle’ (explained below). When entropy is lower than a certain threshold, and a goal has not been found, planning continues, whereas if a goal has been found the corresponding action is performed in the environment. If entropy is above the threshold then the control is passed to the exploration component that selects the action to perform in the world.

#### 1.2.2 Functioning

The functioning of the model is summarised in Algorithm 1. The model experiences multiple trials of the task (lines 1-3 of the algorithm): 60 trials (20 colour triplets) with the goal set to ‘achieve a correct feedback’ (this reflects the target experiment [15]) and 60 trials (other 20 colour triplets) with the goal set to ‘achieve an incorrect feedback’ (as explained below, these additional trials are used to produce a prediction). Each trial of the task lasts for a certain number of discrete time steps (here 15). Each trial involves four phases of functioning of the architecture: the planning phase, (possibly) the exploration phase, the action execution phase, and the learning phase.

**Algorithm 1** Pseudo-code of the model functioning.

1: **loop** VisuoMotorTrials ∈ {1, 2, …, 120}

2:  **if** (VisuoMotorTrials ≤ 60) **then** Goal ← AchieveCorrectFeedback

3:  **else** Goal ← AchieveIncorrectFeedback

4:  EntropyThreshold ← EntropyMax, Planning ← TRUE, Action = NULL

5:  InitialState ← Observe(Environment)

6:  **while** Planning **do**                ⊳ Planning phase

7:   ForwardSampling(InitialState)

8:   Entropy ← ComputeEntropy(AssociativeLayerActivation)

9:   **if** (Entropy > EntropyThreshold) **then**

10:    Planning ← FALSE

11:   **else**

12:    **if** (SampledOutcome = Goal) **then**

13:     Action ← SimulatedAction()

14:     Planning ← FALSE

15:    **else**

16:     UpdateGoalAssociativeConnections()

17:     LowerEntropyThreshold()

18:  **if** (Action = NULL) **then**            ⊳ Exploration phase

19:   Action ← ComputeExplorationAction()

20:  PerformActionInEnvironment (Action)       ⊳ Action phase

21:  Outcome ← Observe(Environment)

22:  TrainWorldModel(InitialState, Action, Outcome)  ⊳ Learning phase

23:  **if** (Outcome = Goal) **then**

24:   UpdateGoalAssociativeConnections()

25:  **else**

26:   TrainExplorationComponent(InitialState, Action, Outcome)

At the beginning of each trial the system observes a colour (lines 5). After the colour observation, the model performs a variable number of ‘planning cycles’ (planning phase, line 6). During a planning cycle, which lasts 15 steps as the actual trial (as in [49]), the input layer is activated with the observed colour for the initial 5 time steps and then is switched off. As a consequence, the associative-layer neurons fire in sequence to simulate a possible colour-action-feedback concatenation (line 7).

During one planning cycle, the arbitration mechanism operates as follows. The sequential neuron sampling causes a certain activation (membrane potential) of the neurons of the associative layer, encoding the probability over the hidden causes: this probability distribution is used to compute the entropy at each step, and this entropy is then averaged over the sampling steps forming the whole planning cycle. This average entropy is considered as the measure of the uncertainty of the world model (line 8). If this uncertainty is higher than a certain threshold, the arbitration component stops planning as not enough confident on the knowledge of the world model (lines 9-10). Instead, if the uncertainty is lower than the threshold the arbitration component checks if the sampled sequence has produced a state (‘read out’ in the output layer) that matches the goal (lines 11-14), and if this is the case it stops planning and performs the action in the environment. Instead, if the arbitration component is confident on the world model but the sampling has not produced a sequence that matches the goal, it performs two operations before starting a new planning cycle: first, it updates the goal-associative connections so as to lower the goal-conditioned probability of the wrong sampled sequence (line 16); second, it lowers the entropy threshold of a certain amount to ensure that across the planning cycles the probability of terminating the planning process progressively increases (line 17): this avoids that the model gets stuck in planning. As soon as the planning process terminates, if the model has not found an action that leads to the goal then the action is selected by the exploration component (lines 18-19).

After this, the agent engages again with the environment. In particular, the action selected either by the planning process or by the exploration component is performed in the environment (line 20). Consequently, the environment produces an outcome (correct/incorrect feedback) perceived by the agent (line 21). Based on the observation of the initial state (colour), performed action (finger press), and outcome (correct/incorrect feedback) from the environment, the world model learns (line 22). In particular, it learns the internal representation (hidden causes) of the observations (input-associative connections), the possible time dependencies between them (internal connections of the associative layer), and the generation of the observations (associative-output connections). Moreover, if the performed action has led to actually accomplish the goal in the environment, the goal-conditioned probability of the sampled successful sequence is increased (goal-associative connections; line 24). Instead, if the action failed then only the exploration component is trained to lower the probability of selecting the same action in correspondence to the experienced initial state and goal (line 26).

Note that when a trial starts, the architecture performs a planning cycle to evaluate entropy: this hypothesis is based on the fact that the task is novel. In a more general case where the agent might also encounter familiar tasks a common habit/planning arbitration process might evaluate if a habit is available to solve the task before triggering planning and the planning/exploration arbitration process considered here.

Note also that in case of goal-failure the goal-associative connections are updated during planning to exclude the multiple sampling of the same wrong sequence and action; instead, in the case of goal-achievement such connections are updated after the action is successfully performed in the environment, rather than in simulation during planning: this avoids a training based on the possible false-positive errors of planning (false-negative errors are less likely during planning as the world model learns on the basis of the ground-truth information from the world). The exploration component is instead trained after the failure of the action executed in the world to avoid to repeat the selection of the actions found to be wrong (this mechanism is analogous to the ‘inhibition-of-return’ found in visual exploration, leading to exclude from exploration already explored items [54]); the component is instead not trained in case of success as this would amount to habitual learning not possible in few trials. These hypotheses were isolated through the search of the conditions for the correct reproduction of the target human data of the visuomotor task while fulfilling the challenging constraint that planning has to take place while learning the neural world model.

Based on these mechanisms, at the beginning of the visuomotor test the model tends to sample random neuron sequences within the associative layer as the world model has no knowledge on the environment. The arbitration component thus soon passes the control to the exploration component that decides which action to execute, and this is performed in the environment. With the accumulation of experienced trials, the world model improves by learning the hidden causes of observations (colours, actions, feedback) and the time dependencies between them. This leads the arbitration component to measure a higher confidence in the world model, so planning continues and samples, with a higher probability, the (hidden causes of) colour-action-feedback sequences that actually exist in the world. When a planning cycle simulates an action that predicts a goal achievement in the output layer, and the action is actually successful when performed in the environment, this leads to increase the goal-conditioned probability of sampling such sequence again so that the next time the same colour is encountered the sequence is readily selected by the planning process.

### 1.3 Goal-directed behaviour model: Detailed functioning

#### 1.3.1 The hidden Markov model represented by the world model

This section first illustrates the graphical models corresponding to the *Hidden Markov Models* (HMMs) and the *Partially Observable Markov Decision Processes* (POMDPs) on which planning as inference is grounded, and then explains the particular HMM instantiated by the world model of our architecture. Next the section illustrates the spiking neural network used to implement this world model and links it to the probabilistic aspects of HMMs.

**Fig 3:**
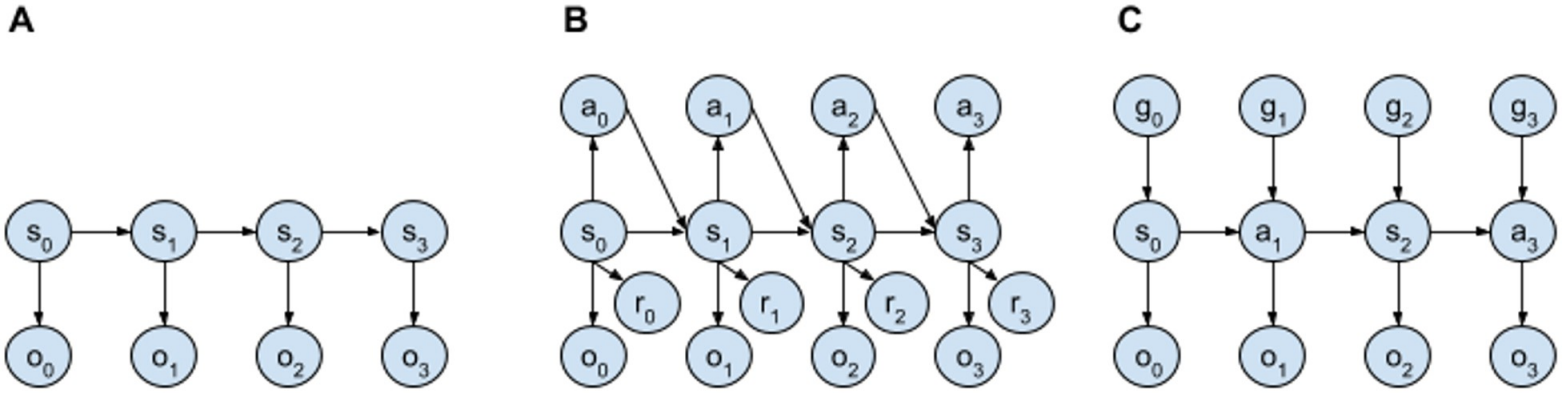
Graphical models of some probabilistic models usable to represent the dynamics of the world in planning systems. Nodes represent probability distributions and directional links represent conditional dependence between probability distributions. (a) Hidden Markov Models (HMMs): these are formed by state nodes ‘s’ and observation nodes ‘o’. (b) Partially Observable Markov Decision Processes (POMDPs): these are also formed by action nodes ‘a’ and reward nodes ‘r’ (different versions of these models are possible based on the chosen nodes and their dependencies). (c) The HMMs considered here, where the planner knows the currently pursued goal ‘g’ and observes not only states but also actions (note that the task considered here involves a sequence of independent state-action-state experiences).

Fig 3 shows a HMM [49, 55] represented through a *graphical model*. A HMM assumes that the agent cannot directly access the world states (they are ‘hidden’ to it) but only infer them on the basis of noisy observations. In particular, the model represents the world states with a different probability distribution, over the possible *hidden causes*, for each time step. The state probability distribution at each time step is assumed to depend only on the state of the previous time step (*Markov property*); the probability distribution over observations is assumed to depend only on the current state. An agent can use a HMM representing the world dynamics to internally simulate possible sequences of states that the environment might traverse, e.g. to represent the places seen while moving through a corridor or the positions of a displaced object.

Building on HHMs, POMDPs again assume that the agent can access the states of the world only indirectly through noisy sensors (they are ‘partially observable’) but they also consider the agent’s behaviour, in particular the probability distributions of actions at different times. Action probability distributions are conditioned on the internal representations of states (thus forming probabilistic *policies*), and over perceived *rewards*. Rewards are considered as additional observations and assumed to depend on other events such as the world states (different models can make different assumptions on rewards). POMDPs can be used to implement planning by conditioning probability distributions on high rewards (or on a reached goal state), and then by inferring the probability distributions of the state-action sequences causing them with a high likelihood (*planning as inference*, [24–26]).

A HMM considers the hidden causes of world states, *h*_*t*_, and observations of them, *o*_*t*_, as random variables at the time steps *t* ∈ {0, 1, …, *T*} forming the sequences *H* = {*h*_0_, *h*_1_, …, *h*_*T*_} and *O* = {*o*_0_, *o*_1_, …, *o*_*T*_}. The joint probability of these sequences can be expressed and factorised as follows given the assumptions on the probability independencies of the model shown in Fig 3A:

$$\begin{array}{c} p\left(H,O\right)=p\left(h_{0}\right)\cdot p\left(o_{0}\right)\cdot \prod_{t=1}^{T}[p\left(o_{t}|h_{t}\right)\cdot p\left(h_{t}|h_{t-1}\right)] \end{array}$$ (1)

This formula highlights the two key elements of the HMM, namely the *generative model* of how the world states (hidden causes) cause the observations, *p*(*o*_*t*_|*h*_*t*_), and the *prediction model* of how a world state causes the following state *p*(*h*_*t*_|*h*_*t*−1_) (in the neural implementation of the HMM we will equivalently consider the probabilities *p*(*o*_*t*_|*h*_*t*−1_), and also *p*(*h*_*t*_|*o*_*t*−1_), to follow the general rule of physical causality for which the current state of any part of the neural network and of the world can depend only on the past state of other parts of the network or the world).

The HMM has parameters *θ* that are adjusted on the basis of collected data (observations) so that the probability distribution *p*(*O*|*θ*) converges towards the empirical distribution from the world, p*(*O*):

$$\begin{array}{c} \theta^{*}=\underset{\theta }{\text{arg}\;\text{min}}\;DL(p^{*}\left(O\right)\|p\left(O|\theta \right)) \end{array}$$ (2)

where *DL*(.‖.) is the Kullback-Leibler divergence between the two distributions and θ* are the searched optimal parameter values of the model. This problem cannot be solved in closed form and so θ* are commonly searched numerically, in particular through an *expectation-maximisation* (EM) algorithm. Here we refer to how this is done in versions of HMMs [49, 56] most similar to the neural implementation of HMMs considered here. For these models, the EM algorithm converges towards the solution by alternating an estimation step (E-step) and a maximisation step (M-step): broadly, the E-step samples a sequence of specific values of the hidden causes, *H*′, based on the posterior distribution *p*(*H*|*O*′, *θ*) dependent on the actual observations *O*′; the M-step adjusts *θ* to increase *p*(*H*′|*O*′, *θ*). In the E-step, the sampling of *H*′ given *O*′ can be approximated by *forward sampling* [57], i.e. by sampling the *h*_*t*_ distributions in sequence, staring from *h*_0_, given the $\left\{o_{0}^{\prime },\;o_{1}^{\prime },\;,...\;o_{t}^{\prime }\right\}$ values observed until *t*.

#### 1.3.2 The spiking neural-network world model

The neural implementation of the world model instantiating the HMM is based on two learning processes. The first process, involving the input-associative connections, learns the hidden causes of different observations as probability distributions of the spikes of the neurons of the associative layer. The second process, involving the connections internal to the associative layer, learns the temporal dependencies between the hidden causes of observations as conditional probability distributions of the spikes of the neurons of the associative layer taking place at succeeding time steps.

The membrane potential of each neuron of the associative layer reflects the activation that would result from the typical connectivity pattern of cortex and other areas of the brain, formed by neurons that reciprocally inhibit each other through inhibitory interneurons. This connectivity pattern tends to keep a constant overall firing rate of the layer. In detail, the membrane potential *u*_*k*_ of a neuron *k* of the model is:

$$\begin{array}{c} u_{k}\left(t\right)={\hat{u}}_{k}\left(t\right)-i\left(t\right) \end{array}$$ (3)

where *i*(*t*) is the common inhibition received by all neurons caused by the inhibitory interneurons to which they project (this inhibition process is abstracted with a *soft-max* function, see below), and ${\hat{u}}_{k}\left(t\right)$ is the total activation received from other neurons:

$$\begin{array}{c} {\hat{u}}_{k}\left(t\right)=\sum_{i=1}^{I}w_{ki}\cdot s_{i}\left(t-1\right)+\sum_{g=1}^{G}w_{kg}\cdot s_{g}\left(t-1\right)+\sum_{a=1}^{A}w_{ka}\cdot s_{a}\left(t-1\right) \end{array}$$ (4)

where *w*_*ki*_ are the input-associative connection weights, *w*_*kg*_ are the goal-associative connection weights, *w*_*ka*_ are the internal associative connection weights, *s*_*i*_(*t*), *s*_*g*_(*t*), and *s*_*a*_(*t*) are the incoming spike signals (*s* ∈ {0, 1}) from the neurons of respectively the input, goal, and associative layer. In the simulations reported in the paper, we also added a Gaussian noise (standard deviation *ν*) to the membrane potential ${\hat{u}}_{k}\left(t\right)$ of associative and output neurons to check the robustness of the model: this did not alter the results with respect to the model not encompassing such noise.

We then assume, as in [49, 58], that the firing rate *v*_*k*_(*t*) of a neuron *k*, reflecting its spiking probability, is exponentially dependent on the membrane potential:

$$\begin{array}{c} v_{k}\left(t\right)=v\cdot e^{u_{k}\left(t\right)} \end{array}$$ (5)

where *v* is a constant scaling the firing rate. This implies the following dependency of the neuron firing rate on the activation from other neurons and on the inhibition from the common inhibition:

$$\begin{array}{c} v_{k}\left(t\right)=v\cdot e^{\left({\hat{u}}_{k}\left(t\right)-i\left(t\right)\right)}=v\cdot \frac{e^{{\hat{u}}_{k}\left(t\right)}}{e^{i(t)}}=v\cdot \frac{e^{{\hat{u}}_{k}\left(t\right)}}{\sum_{l=1}^{L}e^{{\hat{u}}_{l}\left(t\right)}} \end{array}$$ (6)

where *v* now shows to be the total constant firing rate of the population and *i*(*t*) is assumed to be:

$$\begin{array}{c} i\left(t\right)=ln\sum_{l=1}^{L}e^{{\hat{u}}_{l}\left(t\right)} \end{array}$$ (7)

The spiking models we are considering [38, 49] were implemented by assuming continuous time and an inhomogeneous Poisson process to generate the timing of the spikes. However, here we used the version of the model proposed in [38] that considers discrete time steps, a time-binned binary Excitatory Postsynaptic Potentials (EPSP), and a winner-take-all competition to generate a spike at each step. Although having less biological features, this simpler version of the model facilitates the analyses and derivations and at the same time preserves (and possibly strengthens) the probabilistic interpretability of the spiking networks considered. By assuming *v* = 1, Eq 6 becomes a *soft-max* function that abstracts a lateral inhibition-based winner-take-all neural competition. Indeed, now the layer constant total firing is $\sum_{k=1}^{K}v(k)=1$ and *v*(*t*) can be interpreted as *v*(*t*) = *p*_*t*_(*k*), with *p*_*t*_(*k*) being a categorical probability distribution indicating the likelihood that the neuron with index *k* fires a spike at time *t* while the other neurons remain silent. Following [49], the neurons also had a refractory period *r* obtained by subtracting from *u*_*k*_(*t*) a value decaying exponentially at each step *t* (*t* = 0, 1, 2, …) as $r=r_{0}\cdot exp(\frac{-t}{\tau })$ (where *r*_0_ = 1.1, *τ* = 9.5). This feature revealed very important to allow the emergence of groups of neurons encoding the input patterns as latent causes. The output layer, receiving afferent connections from the associative layer, is formed by a set of neurons behaving as the associative layer neurons.

The weights of the connections linking the input-associative layers, the associative-output layers, and the associative neurons between them, are updated through a Spike-Timing Dependent Plasticity (STDP) rule [59–62]. In particular, we used the following STDP learning rule from [38, 49] to update a connection weight *w*_*post*,*pre*_ linking the pre-synaptic neuron *pre* to the post-synaptic neuron *post*:

$$\begin{array}{c} \Delta w_{post,pre}\left(t\right)=\zeta \cdot s_{post}\left(t\right)\cdot (e^{-w_{post,pre}}\cdot s_{pre}\left(t-1\right)-c) \end{array}$$ (8)

where *ζ* is a learning rate parameter, Δ*w*_*post*,*pre*_ is the size of the connection weight update, *s*_*post*_(*t*) and *s*_*pre*_(*t* − 1) are respectively the spike activations (*s* ∈ {0, 1}) of respectively the post-synaptic neuron in the current time step and the pre-synaptic neuron in the last time step, and *c* is a constant (*c* ∈ [0, 1]). The learning rule operates as follows. The rule updates the weight only when the post-synaptic neuron fires (*s*_*post*_(*t*) = 1). When this happens, but the pre-synaptic neuron does not fire (*s*_*pre*_(*t* − 1) = 0), then *w*_*post*,*pre*_ decreases of −*ζ* ⋅ *c*. This leads the post-synaptic neuron to form negative connections with all the pre-synaptic neurons that tend to not fire before it fires. Instead, if the pre-synaptic neuron fires before the post-synaptic neuron (*s*_*pre*_(*t* − 1) = 1), then *w*_*post*,*pre*_ increases if $c<e^{-w_{post,pre}}$ and decreases otherwise. This implies (as it can be seen by solving for Δ*w*_*post*,*pre*_(*t*) = 0 and setting *s*_*post*_(*t*) = 1 and *s*_*pre*_(*t* − 1) = 1) that *w*_*post*,*pre*_ will tend to converge to the positive point $w_{post,pre}^{*}=-ln\left(c\right)$ reached when $e^{-w_{post,pre}}=c$. Overall, for a given neuron the rule thus tends to form positive incoming connections from neurons that fire just before it fires, and negative connections from all other neurons.

The connections that the model learns are the means through which the system implements conditional probabilities. For example, initially the associative units *k*, each representing possible hidden causes of observations, tend to fire with a certain prior probability distribution, say *p*(*k*). The formation of input-associative connections allows an observation *i* to generate the posterior conditional probability distribution *p*(*k*|*i*) that for example implies an increased probability of selection of the hidden cause *k*.

Within the associative layer, the learning rule leads to form a connectivity that supports a sequential activation of the neurons encoding the hidden causes of the observations, where the sequence reflects the temporal order in which the observations, reflecting the world states, are experienced by the HMM. The reason is that once the hidden causes are formed, based on the input-associative connections, then they tend to fire in sequence under the drive of the observations. As a consequence, the learning rule leads each associative neuron to connect with the associative neurons that fired before it and to form negative connections with those that did not fire before it. In this way, the connections within the associative network tend to form chain-like neural assemblies. These connections are hence able to represent the temporal dependencies between hidden causes, for example between *a* and *k* corresponding to two succeeding observations, as conditional probabilities *p*(*k*|*a*). Importantly, if the system observes different events following the initial observation of the trial (e.g., different actions and different outcomes after a certain initial colour), the world model will exploit its stochastic neural processes to represent such possible alternative sequences of events. This is at the core of the architecture’s capacity to internally simulate alternative courses of actions and events and hence to plan in a goal-directed manner.

The same learning rule is also used to train the associative-output connections. Initially, the output layer expresses a probability distribution, say *p*(*o*), that tends to be uniform and so when sampled it generates unstructured observations. With learning, the world model strengthens some connections between the spiking sequences sampled within the associative network and the observations activating the output layer. When the associative-layer world model samples an internal sequence of spikes, this leads to generate the observations on the basis of the reconstruction probability *p*(*o*|*k*).

When the planning process has to generate an action to perform, or a predicted feedback to compare with the goal, the generated event at the output layer is considered to be the one that fired the most during the planning cycle. In cases where the system should generate sequences of events involving multiple actions and predicted states, one should consider other ‘reading out’ mechanisms, for example one where an event is generated each time the units encoding it fire a minimum number of spikes in sequence.

The goal-associative connection weights are updated on the basis of the failure to achieve the goal during planning and in the case of success when the action is performed in the environment. The weight update is done on the basis of the following reinforcement learning rule:

$$\begin{array}{c} \Delta w_{kg}=\eta \cdot m\cdot ET_{k}\cdot (\frac{w_{max}-\left|w_{kg}\right|}{w_{max}})\cdot s_{g} \end{array}$$ (9)

where *η* represents the learning rate, *m* is the pseudo-reward, equal to 1 if the sequence resulted in a successful goal matching (when executed in the environment) and −1 if it resulted in a failure (during planning), *ET*_*k*_ is the Eligibility Trace of the associative unit *k*, equal to 1 for units that have fired at least once during the planning cycle/trial and to 0 otherwise, and *w*_*max*_ is the maximum absolute value that the weight can reach (*w*_*max*_ = 0.5), and *s*_*g*_ is the activity of a goal neuron. The goal-associative connections allow the goal *g* to condition the probability distribution over the hidden causes, *p*(*k*|*i*, *a*, *g*). With learning, this allows the goal to condition the probability of the sampled hidden causes sequences so as to increase the likelihood of those that involve the correct action. Moreover, when the goal changes, the model is able to modify the conditioned probability of the sequences so as to increase the probability of sampling a different sequence, based on the same world model, achieving the new desired goal.

#### 1.3.3 Arbitration and exploration components

The arbitration component decides if continuing to plan or to pass the control to the exploration component and/or to perform the action selected by either the planning or the exploration process. The component makes these decisions on the basis of a key information, namely an estimation of the level of knowledge of the world model for the given trial depending on the observed colour. This knowledge is related to the fact that the world model has possibly learnt that some sequences of events (action-feedback) might follow the initial observation. A good level of knowledge means that the probability mass of the distribution *p*_*t*_(*k*|*i*, *a*, *g*) during the planning cycle steps *t* is concentrated on few possible hidden causes. The measure of this knowledge at a given time step *t* can thus be based on the entropy of the probability distribution expressed by the associative layer:

$$\begin{array}{c} H_{t}\left(k|i,a,g\right)=-\sum_{k=1}^{K}p_{t}\left(k|i,a,g\right)\cdot ln\left(p_{t}\left(k|i,a,g\right)\right) \end{array}$$ (10)

where the maximum value of such entropy is *H*_*max*_ = *ln*(*K*) corresponding to a uniform probability distribution where each *k* neuron of the layer has the same probability of firing *p*(*k*) = 1/*K*. The measure of the uncertainty *H* of the world model in a given planning cycle lasting *T* time steps is in particular defined as:

$$\begin{array}{c} H=\frac{1}{T}\sum_{t=1}^{T}(\frac{H_{t}\left(k|i,a,g\right)}{H_{max}}) \end{array}$$ (11)

At the end of each planning cycle, the arbitration component computes *H*, compares it with an entropy threshold *H*_*Th*_(*t*), compares the action-outcome *z* with the pursued *g*, and selects one of three possible functioning modes of the architecture:

- *H* < *H*_*Th*_(*t*) and *z* ≠ *g*. The goal-associative connections are updated and a new planning cycle starts.
- *H* < *H*_*Th*_(*t*) and *z* = *g*. Planning stops and the action of the last planning cycle that caused the anticipation of the goal is executed in the world (without activating the exploration component).
- *H*_*Th*_(*t*) < *H*. Planning stops and the exploration component selects the action to perform.

The entropy threshold decreases linearly with each planning cycle so that the exploration component is eventually called to select the action if the planning process fails to reach the goal multiple times:

$$\begin{array}{c} H_{Th}\left(t\right)=\epsilon -\left(f\cdot \delta \right) \end{array}$$ (12)

where *ϵ* is the value to which the entropy threshold is set at the beginning of the trial (and the planning process), *δ* is its linear decrease, and *f* is the number of failed planning cycles performed in the trial.

The exploration component is a neural network formed by two layers. The first is an input layer formed by 6 neurons encoding the elements of the Cartesian product between the possible 3 colours and 2 goals. The second is an output layer formed by 5 neurons representing the possible actions, receiving all-to-all connections from the input layer. When the exploration component is called to select the action, the input layer is activated according to the current colour-goal combination (hot-vector activation), the activation potential of the second layer units is computed as usual as the sum of the weighed inputs, and an action is chosen on the basis of a *soft-max* function (Eq 6). When the action leads to a negative reward (−1, received in case of goal missed), the connection weights of the component are updated using the same reinforcement learning rule used for the goal layer (Eq 9). This tends to exclude actions that are not useful for the current state-goal combination, thus fostering exploration. Note that an additional slow-learning component similar to the exploration component might be used to model the formation of habits in experiments involving long learning periods.

### 1.4 Search of the model parameters

The model functioning depends on seven important parameters, indicated in Table 1. We searched the best values of those parameters by fitting the model behaviour to the corresponding data of the human participants. In particular, we randomly sampled and evaluated 100,000 parameter combinations. For each combination, we recorded and averaged the behaviour of 20 ‘simulated participants’, in particular their performance in the 20 trials for each of the stimuli S1, S2, and S3, and the average over colour of the reaction times (this because the original data on the reaction times of humans were not separated). Such three performance datasets and one reaction-time dataset were compared with the corresponding average data from 14 human participants through a Pearson correlation coefficient *R*_*d*,*m*_ computed as:

$$\begin{array}{c} R_{d,m}=\frac{C_{d,m}}{\sqrt{V_{d}*V_{m}}} \end{array}$$ (13)

where *C*_*d*,*m*_ is the covariance between the data from humans, *d*, and data from the model, *m*, and *V*_*d*_ and *V*_*m*_ are their respective variances. In particular, the coefficient was computed separately for the different data sets (performances and reaction times) and then averaged.

**Table 1:** Parameters identified with the grid search technique. In particular, parameter names, minimum and maximum range, and values found by the search.

Name	Range min	Range max	Found value
STDP learning rate (*ζ*)	0.1	1.0	0.96
STDP threshold (*c*)	0.1	1.0	0.67
Planner learning rate (*η*)	0.001	1.0	0.008
Softmax temperature (*τ*)	0.01	0.1	0.02
Neural noise (*ν*)	0.01	0.1	0.02
Entropy max threshold (*ϵ*)	0.3	1.0	0.74
Entropy decrease (*δ*)	0.01	0.2	0.12

The range of the parameters explored by the search, and the best parameter values that it found, are shown in Table 1. The best parameter values, that had a correlation coefficient of 0.72, were used in all the simulations illustrated here.

## 2 Results

This section illustrates the behaviour and functioning of the model when tested with the visuomotor learning task proposed in [15] and described in Sec 1.1.

### 2.1 Behavioural analysis

**Fig 4:**
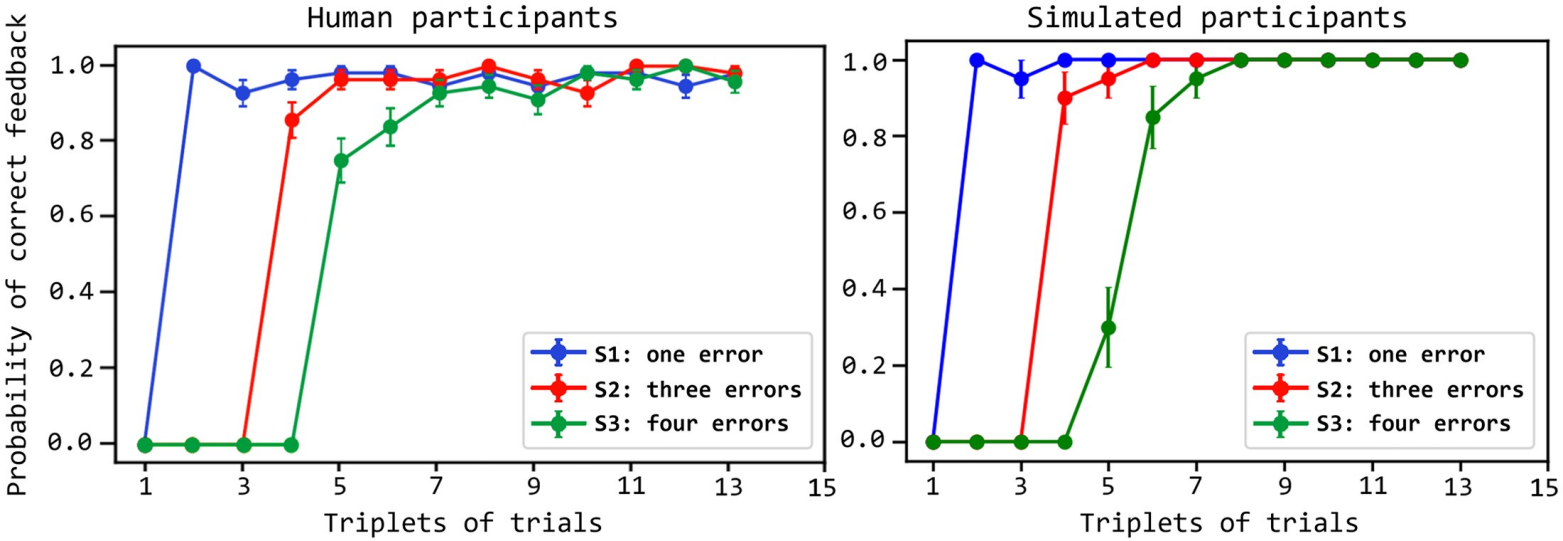
Comparison of the performance of the human and simulated participants. The performance (y-axis) is measured as the proportion of correct feedback over the trial triplets (x-axis), plotted separately for the three different colour stimuli (S1, S2, S3). Curves indicate the values averaged over 14 human participants and 20 simulated participants; error bars indicate the standard error. The data of human participants are from [48].

Fig 4 shows that the model exhibits a performance similar to the human participants by comparing the probability of correct responses in repeated trial triplets for 14 human participants (from [15]) and 20 simulated participants (obtained with different seeds of the random-number generator). The performance of the model is similar to the human one for stimuli S1 and S2 whereas it is slightly lower for S3. Once the model finds the correct action for a stimulus, when it encounters such stimulus again it reproduces the correct action with a high probability similarly to the humans. The architecture takes more cycles to converge to such a high probability for S3 than for S1 and S2 because the planner has a larger number of wrong sequences to explore and so has a higher probability of wrongly anticipating a correct feedback. This problem is less impairing for S1, and in part for S2, involving fewer wrong sequences during planning.

**Fig 5:**
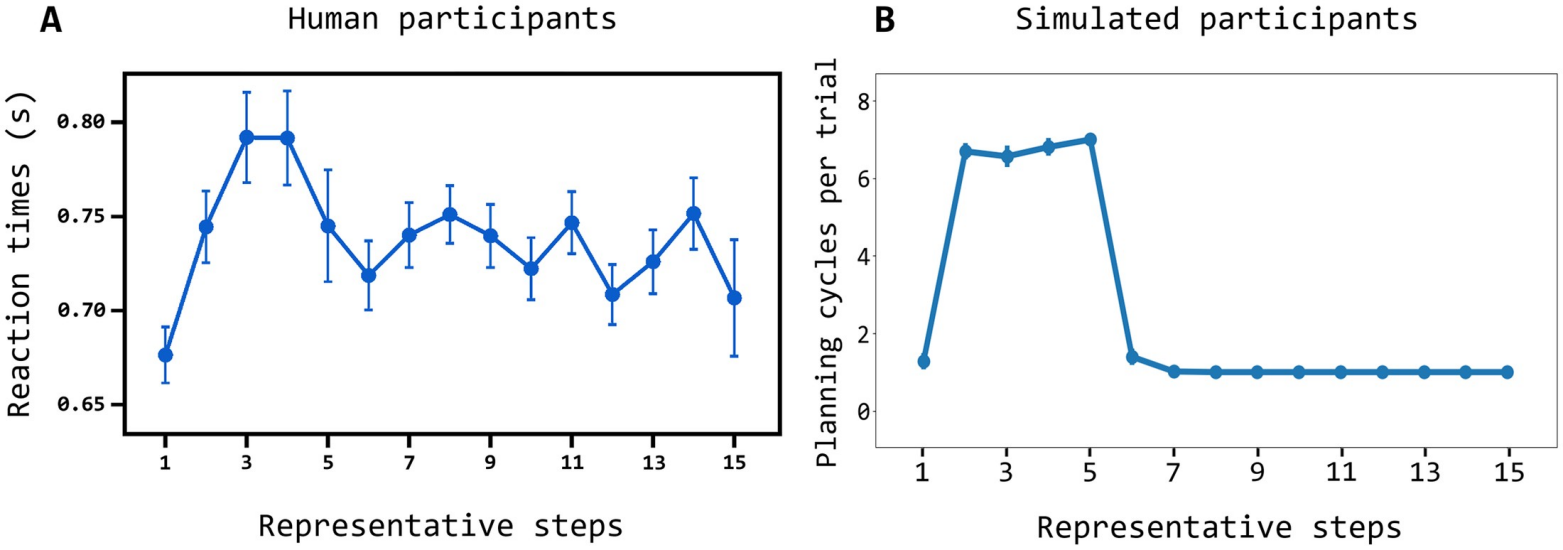
Comparison of the reaction times of the humans and simulated participants. (A) Reaction times of human participants averaged over S1, S2, and S3 (y-axis) for the ‘representative steps’ ([48]; x-axis); the ‘representative steps’ allow the alignment of the reaction times of the three stimuli so as to separate the exploration phase (first 5 steps) and the exploitation phase (6 steps onward); to this purpose, the reaction times for S1 obtained in succeeding trials from the first onward is assigned the steps (used to compute the averages shown in the plot) ‘1, 2, 6, 7, …’, whereas S2 is assigned the steps ‘1, 2, 3, 4, 6, 7, …’, and S3 is assigned the steps ‘1, 2, 3, 4, 5, 6, 7, …’; data are taken from [48]; (B) Reaction times of the model, measured as number of planning cycles performed in each trial, plotted in the same way as done for humans. Error bars indicate mean standard errors.

Fig 5 compares the reaction times of the same human participants (from [15]) and the simulated participants considered in Fig 4. The reaction times of the model are produced by these processes. The arbitration component decides to implement a different number of planning cycles, each involving the generation of colour-action-feedback sequences, depending on the knowledge stored in the world model. If a larger number of planning cycles is performed, this results in longer reaction times. As shown in the graph, the reproduction of the human reaction times is particularly interesting and challenging as it has an inverted ‘U’ shape. This shape is reproduced and accounted for by the model on the basis of the following processes. In the first trials, for each stimulus the entropy (uncertainty) of the world model is high as the associative layer expresses a rather uniform probability distribution. Indeed, the component has still to identify the hidden causes of stimuli and actions, so the neurons encoding them tend to spike at a similar rate. As the entropy is high, the arbitration component tends to soon pass the control to the exploration component and so the reaction times are low in the initial trials. In the following trials the world model forms representations of the experienced colour-action-feedback sequences and so it assigns to them a higher posterior probability with respect to other patterns. The arbitration component thus tends to compute a lower entropy, the architecture plans for longer, and the reaction times get longer. During this planning process, the associative component tends to sample the learnt sequences with a high probability conditioned to the observed colour. If none of the sequences leads to predict an event that matches the pursued goal through the output layer, the probability of such sequences is however decreased under the conditioning of the goal; the control is thus passed to the exploration component. When, during these trials, the action performed in the world manages to produce the desired goal, the world model learns the corresponding sequence and assigns to it a high posterior probability. When the colour of such sequence is observed again, the sequence is sampled with a higher probability and results in a successful outcome-goal match. The arbitration component stops planning and the action is performed in the world. The reaction times thus become short again. Overall these processes reproduce the inverted ‘U’ shape of the reaction times similar to the one observed in humans.

### 2.2 Model internal dynamics

**Fig 6:**
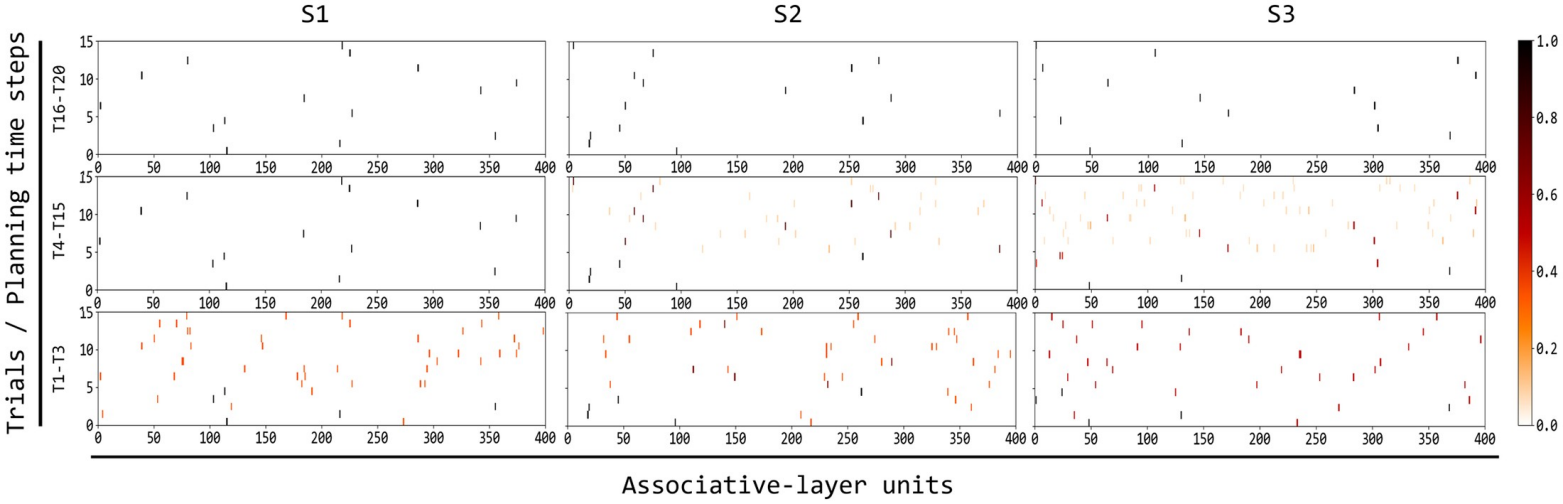
Evolution of the spiking activity of the associative layer units while planning, across the experiment trials. To best interpret the figure, recall that: 15 planning cycles formed one planning sequence (forward sampling), a variable number of planning sequences was generated in one trial for a given colour, 3 trials for the different colours formed a triplet, 20 triplets formed the whole test. The figure shows data collected while the model planned during the trials of the experiment related to each colour, from trial one (T1) to trial 20 (T20). Each column of graphs corresponds to a different colour stimulus, respectively S1, S2, and S3. For each of the nine graphs, the x-axis indicates the indexes of the 400 neurons of the associative layer, and the y-axis indicates the 15 planning cycles of the planning sequences produced in each trial (in each graph the planning cycles progress from bottom to top). Each graph in particular reports the spikes of each neuron for multiple trials (T1-T3 for the bottom row of graphs, T4-T15 for the middle row, and T16-T20 for the third row) and for the multiple planning cycles of those trials: the colour of each little line indicates the proportion of spikes of the corresponding neuron during those trials and cycles.

Fig 6 shows how the activation of the associative layer during planning triggered by the different colours evolves across the succeeding trials of the test due to the increasing knowledge acquired by the world model and by the goal bias. In the initial phases of learning (trials T1-T3 for S1, S2, and S3), the prior probability of activation of the neurons of the associative layer tends to be quite uniform, thus resulting in a random spike sampling of the neurons still not encoding in a sharp way the hidden causes of different colours, actions, and outcomes. This means that the model has not yet identified specific hidden causes of the observations, the temporal relations between them, and the correct colour-action-feedback sequences associated to the three colours.

Based on the observations of the world, the STDP rule acting on the input-associative connections leads the associative layer (world model) to form an internal representation of the hidden causes of the observations, namely of the colours, actions, and feedback. At the same time, the STDP plasticity internal to the associative layer leads it to form a HMM that represents in an increasingly accurate fashion the time-related probabilistic dependencies between the discovered hidden causes. Finally, while possible sequences are encoded by the associative layer, starting from the observed colour, the STDP acting on the goal-associative connections progressively increases the probability of sampling sequences that lead to achieve the goal and to decrease the probability of the sequences that fail to do so.

The effect of these plasticity processes can be seen in the figure graphs (Fig 6). With respect to S1 (three graphs at the left), a population of neurons encoding the correct colour-action-feedback emerges during the initial trials (T1-T3 graph) and later manifests with a sharp activation (T4-T15 graph). For colours S2 and S3 (respectively second and third column of graphs) a successful population of neurons encoding the correct colour-action-feedback takes longer to emerge: during trials T4-T15 (see related graphs) various neural populations fire with a certain probability, and only in trials T16-T20 one stable population encoding the correct sequence linked to the colour emerges. Importantly, during these learning process the world model, which tends to record any aspects of the world dynamics independently of the fact that it is useful to pursue the current goal or not, also learns sequences leading to an incorrect feedback. The next section shows how this knowledge might become useful to accomplish other goals.

**Fig 7:**
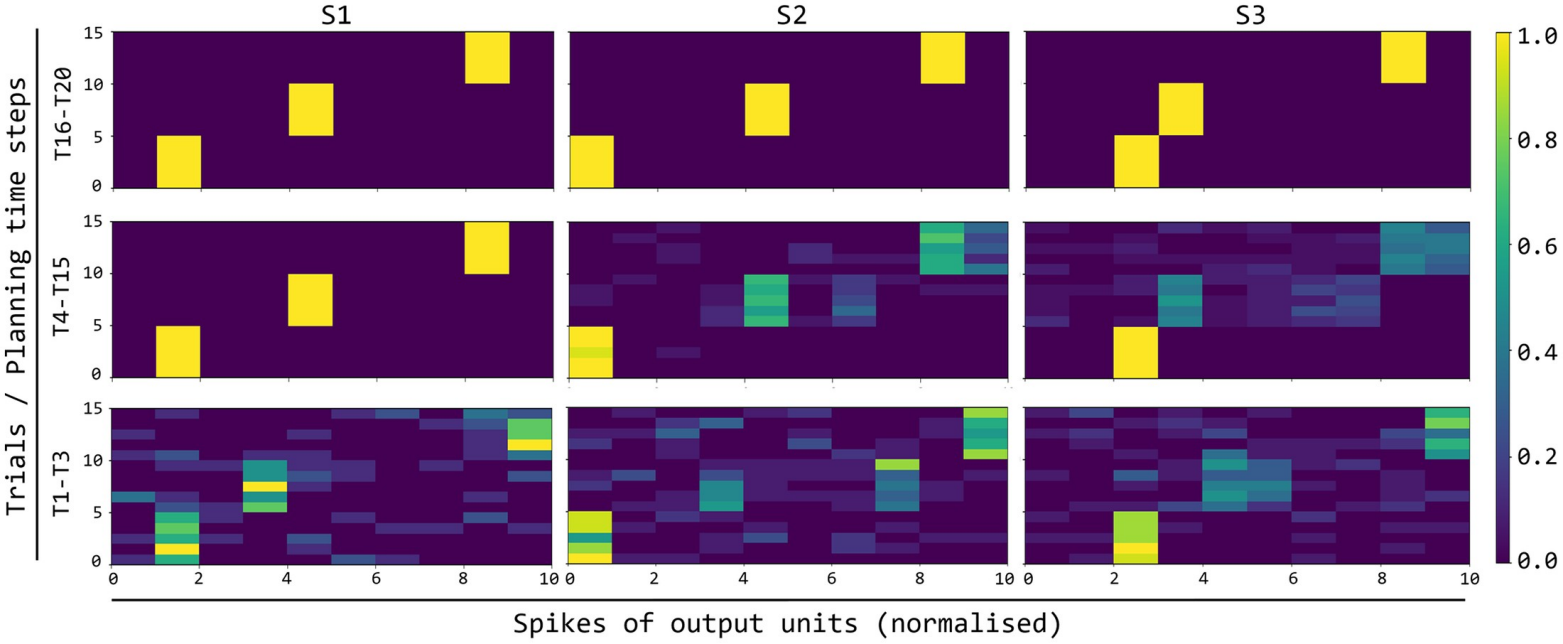
Evolution during trials of the activation of the output layer units encoding the predicted observations and actions. The three columns of graphs refer to the three colour stimuli; the three rows of graphs correspond to different succeeding sets of trials of the task (T1-T3, T4-T15, T16-T20). Each of the nine graphs shows the activation of the 10 output units (x-axis: units 1-3 encode the three colours, units 4-8 encode the 5 actions, and units 9-10 encode the correct/incorrect feedback) during the 15 steps of each trial (y-axis). The colour of the cells in each graph indicates the activation (normalised in [0, 1]) of the corresponding unit, averaged over the graph trials (e.g., T1-T3) and the planning cycles performed within such trials.

Fig 7 shows, with analogous graphs, how the activation of the output layer during the planning trials evolves in time due to the increasing knowledge acquired by the world model. The firing of the output layer during planning expresses the predictions of the events (colours, actions, and feedback) that might happen starting from the observed trial colour. Such predictions are based on the simulation of the possible evolution of the world events based on the HMM instantiated by the associative layer. Regarding S1 (left three graphs of the figure), during the first trials (T1-T3) the world model has no or little knowledge on the dynamics of the world, and so the activation of the units in the output layer reflect a uniform probability distribution leading to random predictions of the trial events. With additional experiences of trials involving S1 (T4-T15), the world model starts to learn to represent the trial events and, under the conditioning of the current goal, to assign a high probability to the correct colour-action-feedback sequence. As a consequence, the probability distribution of the output layer starts to correctly predict such correct sequence.

During trials T4-T15 and T16-T20 the same process happens for the correct sequences of the two colours S2 and S3. Also for these stimuli, towards the end of all trials (T16-T20) the probability distribution expressed by the output layer, conditioned to the associative layer activation, has converged to a probability close to 1 for the correct sequences.

**Fig 8:**
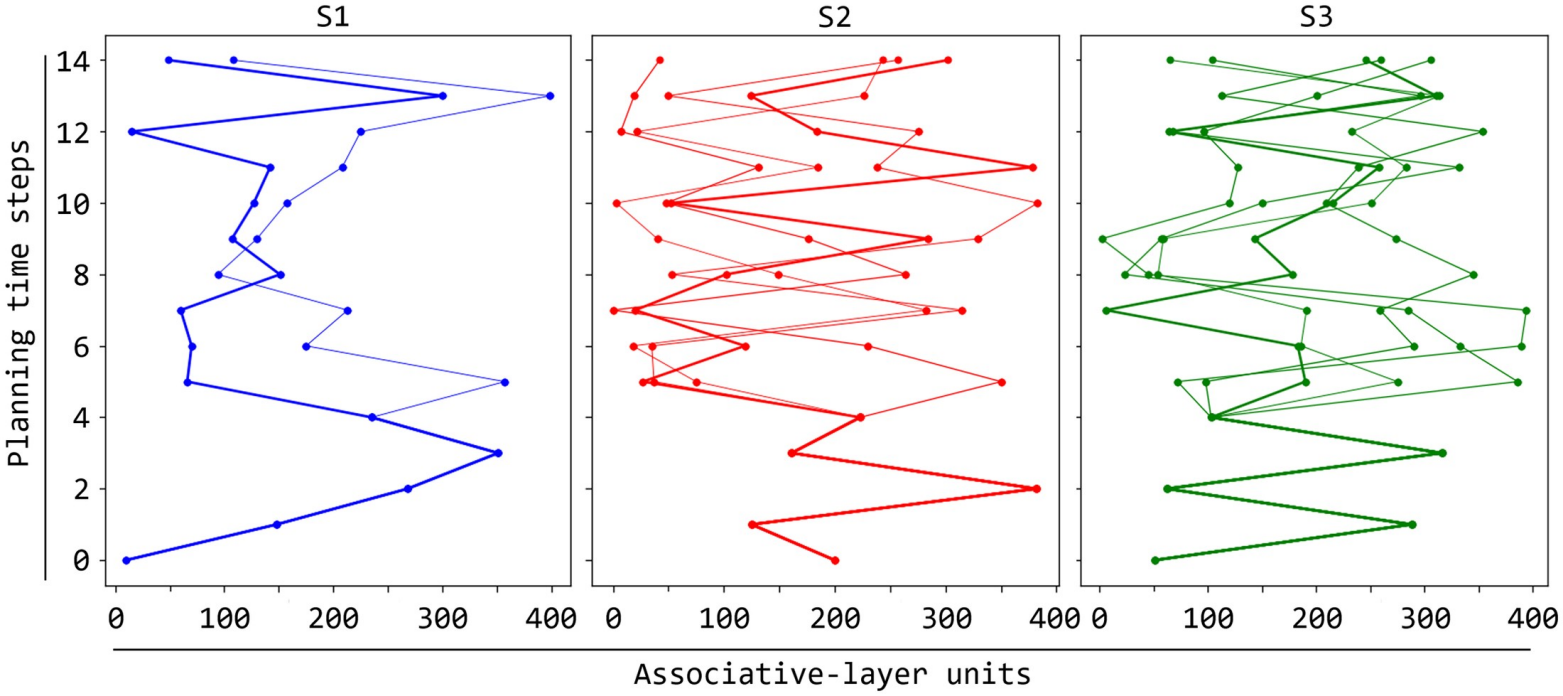
Possible neural trajectories simulated by the model during planning. The three graphs show different neural trajectories that the associative component can generate for respectively the three colours S1, S2, and S3. For each graph, the x-axes indicates the associative neurons and the y-axis the planning time steps and a dot indicates that the corresponding neuron was active. The bolder curve within each graph marks the correct trajectory for the pursued goal ‘correct feedback’.

Fig 8, showing the neurons of the associative layer spiking in sequence during repeated planning cycles, demonstrates how *emergent generativity* (Sec ‘Introduction’) allows the model to imagine different possible future action-outcome trajectories in correspondence to the same colour stimulus. The figure also highlights the trajectory that leads to match the ‘success’ goal. To collect the shown data, we let the model learn until trial T7 for each colour to ensure that it could learn several possible trajectories for it. After this training, we turned off the goal layer to avoid any bias of the associative layer, and let the model perform 400 planning trials for each colour. In this condition, the associative layer responds to the *same* colour by triggering the spikes of *different* possible neuron sequences encoding different colour-action-feedback sequences. Importantly, the figure shows how, when the simulation reaches a ‘branching point’ after the activation of the neurons encoding a certain colour, the stochasticity of neurons at the low-level is amplified by the competition between rival neural populations at a higher-level and this allows the model to imagine different possible actions to perform and feedback to receive. This generativity process supports the ‘cognitive’ exploration of different possible colour-action-feedback trajectories possibly resulting in a successful matching of the ‘correct feedback’ goal.

### 2.3 Predictions of the model

**Fig 9:**
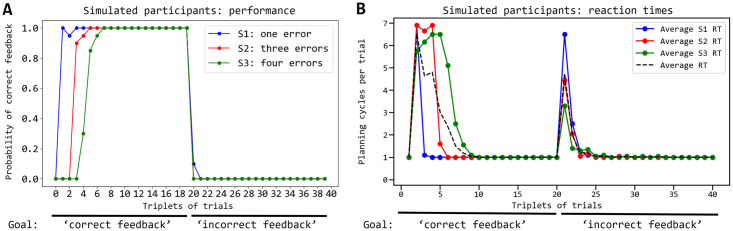
Behaviour of the system when the goal is switched to a new one, averaged over 20 simulated participants. (A) Performance, averaged over the simulated participants, measured as probability of selection of the correct action (y-axis) along the trial triplets (x-axis); the pursued goal is switched from getting a ‘correct feedback’ to getting an ‘incorrect feedback’ at triplet 20. (B) Average reaction times measured during the same experiment shown in ‘A’.

An important advantage of planning is that the world model can store general knowledge on the dynamics of the world and this can be used to accomplish different goals. This is a prototypical feature of goal-directed systems that allows them to rapidly switch behavior if the goal changes (*behavioral flexibility*). It was thus interesting to check to which extent the current architecture preserved this capacity since it incrementally acquires a *partial world model* while solving the visuomotor task (‘partial’ as the solution of the visuomotor task requires the discovery of only the correct colour-action-feedback sequence for each colour, not of all possible sequences). To this purpose, after the architecture solved the task as reported in the previous section, it was given 20 additional trial triplets to pursue the different goal of ‘obtaining an incorrect feedback’ in correspondence to the three colours. Fig 9A shows the results of this test. When the goal is switched, the architecture is able to rapidly change behaviour and choose the sequences that lead to the desired new goal given the colour. What happens is indeed that, under the conditioning of the observed colour, the world model already has the representations of (a) the hidden causes of the possible observations and (b) of the possible sequences with which such observations might be experienced. In particular, since the previous goal unit is now off, the probability of the different sequences tends to be similar, and so the system tends to sample all of them equally during planning. However, this allows the architecture to rapidly discover a sequence that leads to the desired new goal and thus to increase the probability of generating such sequence conditionally to the new goal.

Regarding the reaction times (Fig 9B), the model shows a transient increase of their size in correspondence to the goal switch. This is due to the fact that with the new goal the architecture needs to perform the sampling of some sequences before finding the successful ones. The reaction time is higher for S1 than for S2 and S3 as for it the model has less sequences available to reach the new ‘incorrect feedback’ (instead, the model has exactly one sequence to achieve the ‘correct feedback’ goal for each colour).

These results represent a prediction of the model that might be tested in a future experiment with human participants through a test analogous to the one presented in this section. In particular, the test should measure the dynamics across trial triplets of the performance and reaction times differentiated by the three colours (S1/S2/S3), as shown in Fig 9.

## 3 Discussion

This section discusses the main features of the model by first analysing the results presented in the previous section and then by considering the model general features by also comparing it with the current state-of-the-art probability-based or spiking neural-network models of goal-directed behaviour and planning.

### 3.1 Discussion of the specific results

As shown in Sec 2.1 and in Fig 4, once the model finds the correct action for one colour the probability of correct answers rises steeply, in agreement with what happens with the human participants of the target experiment. Moreover, as in humans, the architecture takes more cycles to converge to such a high probability for S3 because the planner has a larger number of wrong trajectories to explore and so has a higher probability of wrongly anticipating a correct feedback; this problem is less impairing for S1 and S2 involving fewer wrong trajectories that the planning process has to consider. Note how this result, and the one on the reproduction of the reaction times commented below, is not a mere fitting exercise as the architecture reproduces the target data while satisfying a number of biological and ecological constraints, in particular: (a) it solves the task only through goal-directed process, and not through habitual mechanisms as done by previous models of the task [48]: this is requested by the short duration of the task that does not allow habit acquisition; (b) the world model, representing the core of the architecture, relies on spiking-neural mechanisms and biologically plausible circuits; (c) planning takes place while the world model is being acquired, as imposed by the solution of tasks involving new portions of the environment; (d) the model uses an unsupervised learning process.

Fig 5 shows how the model reproduces human reaction times quite accurately as the differences with the target human data are due to some simplifying assumptions of the model. The differences are that the reaction times are above zero for the human participants and close to zero for the model, and that in the first trial they are lower than those of the steady-state trials for the human participants whereas they are similar for the model. The reasons of the first difference is that human participants are likely endowed with an additional habitual/goal-directed arbitration mechanism making a decision before the second exploration/exploitation arbitration mechanism considered here is activated, and this increases the reaction times of a certain amount for all trials. The second difference could be explained by the fact that human participants listen to an explanation of the task before solving it and so they likely start the test having already decided that they should not plan in the first trial, which thus has a low reaction time; instead, the model attempts to plan also in the first trial to check if it is able or not to solve the task.

A second result is the model capacity to reproduce the inverted ‘U’ shape of the reaction times exhibited by human participants and to explain it. In particular, the model suggests that: (a) in the initial trials, the world model has learnt no state-action sequences, its entropy is high, and so the arbitration component passes the control to the exploration component: the reaction times are hence short; (b) when the world model has learnt some sequences, but these are wrong, planning implements several cycles to explore such sequences and to lower their goal-conditioned probability, so the arbitration component takes time to pass the control to the exploration component: the reaction times are hence long; (c) when the world model has learnt the correct sequence, entropy is low and thus the planning process samples the correct sequence with a high probability, obtains a successful matching of the goal, and triggers the performance of the related action: the reaction times hence become short again. Another model [48] used an entropy-based measure as a means to decide to give control to a goal-directed component or to a habitual component, and reproduced the ‘U’ shape of the reaction times observed in the target experiment considered here. This model was based on a goal-directed component formed by a *Bayesian Working Memory* (a memory of the probabilities of the time-dependent states, of the one-step environment transitions, and of the rewards) and a habitual component (based on *Q-learning*). The model reproduced the ‘U’ shape of reaction times as the sum of two values: (a) the logarithm of the number of items in working-memory, related to the performed trials; (b) the entropy of the action probabilities. The inverted ‘U’ shape of reaction times was obtained by the fact that ‘a’ tends to increase with the accumulation of items in memory while ‘b’ tends to decrease with the diminishing variance of the action probability. In comparison, the model presented here produces the inverted ‘U’ shape as an emergent effect of the change of knowledge of the world model. The empirical and computational implications of the two hypotheses presented here and in [48] deserve further investigations.

Figs 6, 7 and 8 visualise the internal functioning of the model, in particular the activation of neurons that dynamically encode multiple sequences of colour-action-feedback during planning. The figures highlight two relevant features of the model, in particular: (a) its capacity to autonomously form neural internal representations (hidden causes) of the observations at different times and to activate them in sequence: this capacity relies on the used STDP unsupervised learning rule and the features of the model architecture; (b) the sampling done by spikes of the probability distributions expressed by the world model, and the emergent generativity of the architecture (further discussed below): these processes rely on the stochastic nature of the model, allowing it to ‘imagine’ different possible action-feedback sequences in correspondence to a colour stimulus.

Fig 9 shows that once the world model has acquired goal-independent knowledge on the environment dynamics, the architecture can use it to pursue different goals ‘on the fly’, i.e. without the need to further train the world model. This feature is the hallmark of the flexibility of goal-directed behaviour and is shared with the previously state-of-the-art planning-as-inference models relying on spiking neural-networks [39, 42]. However, these models were not used to produce specific empirical predictions as here.

### 3.2 Discussion of the general features of the model

The results also highlight the novelties of the proposed architecture with respect to the current models. A first novelty with respect to the previous models implementing planning as inference based on brain-like mechanisms [38–40] is that our architecture proposes an hypothesis on how organisms might learn the world model while using it for planning. This is a key challenge for planning, as recently highlighted in [46]. The challenge is different from the exploration/exploitation issue in model-free models [4], and requires arbitration mechanisms different from the classic ones used to balance goal-directed and habitual processes [47, 48]. The work [46] highlights how the challenge is made hard by the ‘bad-bootstrapping’ problem, mentioned in the Introduction, for which the world model tends to prematurely converge to sub-optimal solutions due to the biased selection of actions directed to pursue goals. The work also presents a model offering a solution to the challenge based on a suitable balance of the selection of goal-directed and exploration actions. The solution is based on the minimisation of ‘free energy’, which is the pivotal quantity of the *active inference* framework [22], and in particular on probability functions related to the events of interest and a derivative-based optimisation algorithm. On this respect, our model is the first to present a solution to the challenge based on brain-like spiking neural network. In addition, the model presented in [46] gives a principled solution with respect to previous probabilistic models [47, 48], but for now it is applicable only to simple tasks that require the agent to learn the probability function parameters while being given *a-priori* the set of possible hidden causes of observations. Instead, the model presented here is able to autonomously learn the hidden causes of observations based on spiking neural-network mechanisms.

A second novelty of our model with respect to previous models implementing planning as inference based on brain-like mechanisms [38–40] is that it learns the world model on the basis of a biologically plausible *unsupervised* learning mechanism rather than on the basis of a supervised learning algorithm [38, 39] or by using a world model given *a-priori* [40]. This is an advancement for the biological plausibility of planning as inference models. Indeed, from a computational perspective finding the conditions for the successful functioning of such unsupervised learning process, contextually to the solution of the previous problem related to the acquisition of the world model during planning, represented the hardest challenge found in developing the architecture. We now briefly discuss the three main innovations that support the solution.

First, we grounded learning on the STDP unsupervised learning rule proposed in [49]. This rule is ideal to allow the self-organisation of the architecture associative layer leading to form both the neural representations of hidden causes of observations and the temporal dependencies between them, as required by the autonomous learning of the world model through the spiking recurrent network. Given a neuron that fires, the rule tends to increase the afferent connection weights from neurons that have fired in the recent past, and to decrease connection weights from neurons that have not fired: in the presence of a strong lateral inhibition installing a competition between neurons, as it happens in several parts of brain, this mechanism leads to the emergence of cell groups that specialise to maximally respond to specific (possibly delayed) input patterns. Notice how this mechanism has interesting analogies with the learning processes used in rate-based Self-Organising Maps [63, 64].

A second novel feature that allowed the architecture to autonomously learn the world model is the use of a HMM having a relevant difference with respect to those used in other planning-as-inference spiking network models [38, 39]. These models use a world model based on a classic HMM reproducing possible sequences of states but not actions. Instead, the world model used here is based on a HMM that observes sequences of states *and of actions*, respectively produced by the environment and by another component of the architecture (e.g., by the exploration component used here). This has various possible advantages. One advantage, employed here, is that the world model can directly select actions to perform; instead, previous models [38, 39] need an additional mechanism selecting actions on the basis of the state sequence produced by the world model. A second advantage is that for each environment state the world model can suggest the selection of actions that have a potential relevance in that context, rather than any action (this captures the popular idea of *affordance* in cognitive science [65, 66]). A last advantage could be the easier learning (and understanding) of state-action sequences directed to a goal produced by other agents; indeed, the world model would be neutral with respect to the fact that actions are performed by another part of the brain or by another agent.

A third and last novel feature that allowed the architecture to autonomously learn the world model is the explicit representation of the *goal* used to condition the probability distribution expressed by the world model. Previous state-of-the-art models [38, 39] conflated the goal, initial state, and environment conditions into a whole ‘context’ representation. Our representation of goals allows their manipulation independently of other conditions, as shown by the model’s capacity to successfully plan how to reach new goals on the basis of the experience that the world model acquired in other tasks. Moreover, it paves the way to the enhancement of the architecture with mechanisms allowing the autonomous selection of goals.

We now consider what we think to be a very important mechanism used by the model: emergent generativity. Although shared with other previous models, here we aim to explicitly identify its general features and to stress its wide scope and importance. With *emergent generativity* we refer to the property of a spiking neural-network system for which the low-level stochastic events represented by the spikes of neurons are possibly ‘amplified’ by the neural circuitry of the system to actively generate multiple alternative high-level patterns –encoding cognitive contents such as percepts, motivations, thoughts, actions, and plans– useful to support adaptive behaviour. The key ‘ingredients’ of emergent generativity are hence: noisy low-level stochastic units, circuits supporting competitive activation mechanisms, STDP-like unsupervised learning processes, and high-level cognitive processes and behaviours.

Emergent generativity is characterised by two relevant elements. The first element regards ‘generativity’ and involves the stochastic nature of spike sampling that allows the production of *alternative* patterns in correspondence to the *same* input/context. This process is important as the generation of alternative plausible patterns is at the core of search algorithms possibly employed by brain (by ‘plausible patterns’ we mean patterns having a high chance to satisfy some constraints, e.g. ‘images you might see in a certain environment’, or ‘actions you might be able to perform with your body’). For example, generativity can support the search of different courses of action that might lead to a desired goal state starting from a given initial condition. In neural networks, generativity is often based on stochastic elements supporting the generation of novel plausible patterns. Notable examples of these systems are Generative Adversarial Networks (GANs; [67]) and Variational Autoencoders (VAEs; [68]) able to generate new plausible input patterns by drawing sample patterns from prior probability distributions of ‘latent variables’ (hidden causes) and then by transforming them through deterministic neural components trainable with supervised learning (some recent versions of VAEs are also able to learn and generate sequences of hidden causes, analogously to HMMs [69]). These neural systems offer a good intuition on the potential utility of generativity, but within them what we can call the ‘stochastic generative engine’ (meaning the stochastic mechanism at the core of the generation of alternative plausible patterns) is limited to a particular portion of the system, for example the stochastic input sent to the ‘generator’ in GANs or the stochastic ‘bottleneck’ in VAEs. Importantly, such stochastic mechanisms are in contrast with the use of gradient descent algorithms needed to implement supervised learning as they introduce discontinuities preventing differentiation (e.g., VAEs have to use a ‘reparameterization trick’ to allow the gradient information to ‘pass through’ the bottleneck stochastic nodes). Instead, in spiking neural networks each spiking neuron, if endowed with intrinsic stochasticity, represents a ‘micro stochastic generator’ and so the ‘stochastic generative engine’ of the whole system is distributed in each part of the system rather than being confined in specific locations of the architecture as in GANs and VAEs (as mentioned in the Sec ‘Introduction’, this shares analogies with *particle filters* [36, 37]). Although this possibility was not exploited here, it might be explored in future work, for example to support planning at multiple levels of abstraction. The use of stochastic units in all parts of the system requires the use of learning rules not requiring differentiation across neural layers, such as the STDP unsupervised learning rule used here. In this respect, *Boltzmann Machines* and *Restricted Boltzmann Machines* are interesting neural network models that, although now less popular, might be relevant to study systems exhibiting emergent generativity since they are based on an architecture fully based on stochastic neurons and use local unsupervised learning rules [70–72]. The second important element of emergent generativity regards ‘emergence’ and involves the process for which in complex systems, such as the brain, the dynamical interaction of low-level elements can give rise to organised patterns at higher levels [73]. In particular, in the brain, events involving spike neurons at a low (micro) level are amplified by neural mechanisms in order to generate patterns that encode content, such as perceptions, thoughts and actions, at a higher (macro) cognitive level. As shown here, the ‘amplification’ can for example rely on circuits implementing winner-take-all competitions grounded on typical connectivity patterns of the brain micro-circuits, and on unsupervised learning processes relying on the brain spike-timing dependent plasticity (STDP) [49, 74–76]. Interestingly, as mentioned above, these mechanisms are analogous to those used in self-organising neural networks [63, 64]. Importantly, the fact that multiple levels of organisation indeed characterise models as the one presented here becomes apparent if one considers that the support of the probability distributions in spiking networks correspond to the identity of neurons, whereas the support of the probability distributions of percepts, actions, and thoughts corresponds to the states of sensors, actuators, and other neural components. This contrasts with the generativity of standard probability models, as those commonly used in planning as inference, where the support of the used probability distributions directly corresponds to the states of percept, actions, thoughts. In summary, emergent generativity featured by the brain has these advantages: by default, the brain can learn the probability distributions of the hidden causes of any relevant cognitive element, the support (representation) of such distributions, and the probability dependencies between such causes. We speculate that the importance of these advantages might have contributed to lead evolution to endow the brain with spiking neurons rather than with firing-rate neurons (cf. [21, 37, 77]).

## 4 Conclusions

Goal-directed and planning processes can support flexible behaviour based on the use of general-purpose knowledge on the world. In recent years, it has been proposed that planning processes in the brain are based on probabilistic representations of the world and inferences on them. This proposal is very interesting but it encounters the great challenge of explaining how such representations and inferences might be grounded on the actual neural computations of the brain. Recently, some models have been proposed to ground some probability inference mechanisms, such as Hidden Markov Models and Partially Observable Markov Decision Processes, on the spiking stochastic events exhibited by the brain neurons and their connectivity patterns and plasticity mechanisms.

Here we propose a spiking neural-network architecture facing two important problems not solved by the state-of-the-art models bridging planning as inference and brain-like mechanisms, namely the problem of learning the world model contextually to its use for planning, and the problem of learning such world model in an autonomous fashion based on unsupervised learning processes. The architecture has been validated with data from human participants engaged in solving a visuomotor behavioural test that requires the discovery of the correct actions to associate to some stimuli [15]. The architecture has reproduced the target behaviour, has furnished an explanation of the mechanisms possibly underlying it, and has proposed predictions testable in future empirical experiments.

To overcome the two mentioned problems, the architecture proposes two novel mechanisms that the brain might use to solve them. First, it introduces a new arbitration mechanism that leads the model to plan and act to pursue the goal, or to explore to train the world model, on the basis of the knowledge of the world model itself: this knowledge is measured as the entropy of the goal-conditioned probability distribution of future states and actions expressed by the world model. Second, the model is able to autonomously learn the world model by integrating an STDP unsupervised learning rule proposed in the literature [49], with a world model based on a HMM whose observations involve not only world states but also actions, and using a goal representation to condition the probability distribution expressed by the world model.

We acknowledge that the model has various limitations that might be improved in future work. A first one concerns the passage from neurons firing at discrete times to neurons firing in continuous time. This might be done using the inhomogeneous Poisson process used in [49]. Although this would not change the theoretical contribution of the model, it might simplify a comparison of the model functioning with real data from the brain at a finer temporal level with respect to what done here.

A further issue to face would be to use other tasks with respect to the one considered here [15], for example to develop the model to consider tasks requiring longer sequences of states and actions as was done in [38, 39]. The latter works also suggest the interesting possibility of employing the model to control autonomous robots to test its robustness and capacity to scale-up to more complex tasks.

A relevant issue to face in future work concerns the new arbitration mechanism proposed with the model. The entropy measure at the core of such arbitration mechanism is grounded on the probability distribution of neurons. However, the mechanism using such information to arbitrate between planning and exploration is now hardwired. Future work should thus aim to implement this process with neural mechanisms. For example, the entropy measure might be ‘read out’ by an additional neural layer that could then selectively inhibit either the planning or the exploration component.

Another improvement of the model might involve the full development of a habitual component. Here we did not introduce such component as the target experiment covered a short learning time not allowing the formation of habits, so we focused on considering the exploration/exploitation processes involved in the early phases of learning of new tasks. Future work might however also consider the formation of habits, for example by targeting additional experiments involving long ‘over-training’ periods. This could be done with components analogous to the exploration component used here, but using slow reinforcement learning processes to represent the slow formation of habits favouring generalisation. The addition of habit learning processes would also require the introduction of a further arbitration mechanism as those proposed in [47, 48] to harmonise goal-directed and habitual behaviour.

A further possible improvement of the model concerns the treatment of goals. These are now selected externally and represented in a simple way. Goals could instead be represented in more realistic ways, for example through mechanisms mimicking working memory [78], and could be selected in autonomous ways, for example based on motivational mechanisms [72, 79].

A last possible improvement of the model concerns the possibility of testing and constraining the model not only at the behavioural level, as done here (and as also done by previous probabilistic models investigating arbitration mechanisms in goal-directed behaviour, e.g. [47, 48]) but also at the neural level, for example based on data collected on similar experiments [80, 81]. This might for example be done through techniques such as *Representational Similarity Analysis* [82] that uses brain-imaging data to map the components of neural models to areas of the brain that possibly implement analogous functions.

Notwithstanding these limitations and possible improvements, we think the proposed architecture represents a further step towards the realisation of models that implement probabilistic versions of goal-directed processes on the basis of brain-like mechanisms, in particular spiking neurons, competitive circuits, and STDP unsupervised learning rules. In particular, the model contributes to formulate new hypotheses on how the brain might acquire the world model needed for planning in a fully autonomous way while at the same time using it for planning.
